# The Effect of Geometrical Isomerism of 3,5-Dicaffeoylquinic Acid on Its Binding Affinity to HIV-Integrase Enzyme: A Molecular Docking Study

**DOI:** 10.1155/2016/4138263

**Published:** 2016-10-18

**Authors:** Mpho M. Makola, Ian A. Dubery, Gerrit Koorsen, Paul A. Steenkamp, Mwadham M. Kabanda, Louis L. du Preez, Ntakadzeni E. Madala

**Affiliations:** ^1^Department of Biochemistry, University of Johannesburg, P.O. Box 524, Auckland Park, Johannesburg 2006, South Africa; ^2^Council for Scientific and Industrial Research (CSIR), Biosciences, Natural Products and Agroprocessing Group, Pretoria 0001, South Africa; ^3^Department of Chemistry, Faculty of Agriculture, Science and Technology, School of Mathematical and Physical Science, North-West University, Mafikeng Campus, Private Bag X2046, Mmabatho 2735, South Africa; ^4^Material Science Innovation and Modelling (MaSIM) Research Focus Area, Faculty of Agriculture, Science and Technology, North-West University, Mafikeng Campus, Private Bag X2046, Mmabatho 2735, South Africa; ^5^Department of Microbiological, Biochemical and Food Biotechnology, University of the Free State, Bloemfontein, South Africa

## Abstract

A potent plant-derived HIV-1 inhibitor, 3,5-dicaffeoylquinic acid (diCQA), has been shown to undergo isomerisation upon UV exposure where the naturally occurring 3^*trans*^,5^*trans*^-diCQA isomer gives rise to the 3^*cis*^,5^*trans*^-diCQA, 3^*trans*^,5^*cis*^-diCQA, and 3^*cis*^,5^*cis*^-diCQA isomers. In this study, inhibition of HIV-1 INT by UV-induced isomers was investigated using molecular docking methods. Here, density functional theory (DFT) models were used for geometry optimization of the 3,5-diCQA isomers. The YASARA and Autodock VINA software packages were then used to determine the binding interactions between the HIV-1 INT catalytic domain and the 3,5-diCQA isomers and the Discovery Studio suite was used to visualise the interactions between the isomers and the protein. The geometrical isomers of 3,5-diCQA were all found to bind to the catalytic core domain of the INT enzyme. Moreover, the *cis* geometrical isomers were found to interact with the metal cofactor of HIV-1INT, a phenomenon which has been linked to antiviral potency. Furthermore, the 3^*trans*^,5^*cis*^-diCQA isomer was also found to interact with both LYS156 and LYS159 which are important residues for viral DNA integration. The differences in binding modes of these naturally coexisting isomers may allow wider synergistic activity which may be beneficial in comparison to the activities of each individual isomer.

## 1. Introduction

The development of therapeutics against the Human Immunodeficiency Virus type 1 (HIV-1), which causes Acquired Immunodeficiency Syndrome (AIDS), is still an active area of research. HIV-1 has, among other important components, three enzymes that are essential for viral replication and subsequent infection of humans, namely, HIV-1 protease (PROT), reverse transcriptase (RT), and integrase (INT) enzymes [1]. HIV-1 INT catalyses the incorporation of viral DNA into the host cell genome after the viral RNA has been reverse transcribed; this step is crucial in viral replication and makes anti-HIV-1 INT inhibitors an important field of research [2, 3]. The integrase enzyme has three domains, the C-terminal domain (CTD), the catalytic core domain (CCD), and the N-terminal domain (NTD) [4]. The CCD has a conserved catalytic triad, the DDE motif, which consists of the residues ASP64, ASP116, and GLU152 [5]. Within the catalytic core domain of HIV-1 INT is the divalent metal (Mg^2+^ or Mn^2+^) cofactor that deprotonates water for 3′ end processing of viral cDNA, a reaction that involves transesterification [3, 6]. Other amino acid residues (LYS156 and LYS159) are also important for viral DNA binding before further processing can occur [6].

The dicaffeoylquinic acids (diCQAs), a group of plant secondary metabolites, have been found to possess specific and irreversible inhibitory activity against HIV-1 INT making them potent inhibitors [7, 8]. These compounds have been isolated from herbal plants that have been noted for their anti-HIV activity such as* Helichrysum populifolium* [9],* Centella asiatica* [10, 11], and* Aster scaber* [12]. The diCQAs can either bind to the active domain or to a flexible loop near the catalytic site of the enzyme to inhibit viral DNA integration to the host cell DNA [13]. One such diCQA isolated from herbal plants which has been found to be active against HIV-1 INT is 3,5-diCQA, an ester of quinic acid and two caffeic acid moieties substituted at the 3 and 5 positions of the quinic acid [14]. From docking studies of L-chicoric acid and HIV-INT, it can be assumed that the caffeic acid units are the main pharmacophores of the diCQAs [15].

UV-irradiation of the naturally occurring 3^*trans*^,5^*trans*^-diCQA causes the geometrical isomerism of this molecule in the caffeic acid units and gives rise to* cis* isomers: 3^*cis*^,5^*trans*^- diCQA, 3^*trans*^,5^*cis*^-diCQA, and 3^*cis*^,5^*cis*^-diCQA [14, 16, 17]. Isomerism of 3,5-diCQA in the quinic acid unit has been shown to affect its activity against HIV-1 INT [12]; however, the effect of geometrical isomerism in the caffeic acid units has not been investigated for this molecule. The stereochemistry of bioactive compounds is important since different stereoisomers have different pharmacodynamics in humans [18]. This is due to the fact that target biomolecules are stereospecific [19]. Though enantiomers are often of more concern than other types of stereoisomers [20], the effects of regional and geometrical isomers on biological activity still require further investigation. For instance, Farrer et al. found that* trans* platinum complexes were more active against keratinocytes and human ovarian cancer cells than their* cis* counterparts [21]. Furthermore, photoisomerisation of* cis* tetrazoline oxime ethers to the* trans* isomers resulted in a loss of efficacy of these fungicides when used on plants exposed to UV-irradiation [22]. Given this importance of the stereochemistry of bioactive molecules on their activities, we investigated the differences in the binding interactions of HIV-INT and the UV-induced geometrical isomers of 3,5-diCQA.

## 2. Methodology

### 2.1. Ligand

A random conformational search approach was used to find the lowest energy conformer for each isomer. The geometries of the four 3,5-diCQA isomers were then optimised,* in vacuo*, using density functional theory with the B3LYP functional and the 6-311+G (d, p) basis set. The calculations were carried out on the Gaussian09 software package [23]. Optimised structures were visualised using the GaussView05 software.

### 2.2. Molecular Modelling

All molecular modelling was performed using YASARA Structure [24]. The intended receptor crystal structure (PDB code: 1QS4) contained two identical chains, A and B. Chain A was chosen as receptor; however, it was missing residues 141–144. Chain B contained residues 143 and 144 and these residues were inserted into chain A by first superimposing chain B onto chain A and then removing all other chain B residues and finally merging the residues into chain A (all other residues were deleted). The two remaining residues were artificially added (Ile 141 and Pro 142). All residues in the structure, except for residues 140 to 145, were fixed and energy minimization was performed to relax the flexible region. Subsequently, all residues were made flexible and another energy minimization was performed on the entire structure. The resulting structure was used as the receptor in the subsequent molecular docking experiments.

### 2.3. Molecular Docking

Molecular docking experiments were prepared using YASARA [24]. A rectangular box with dimensions 30 Å × 30 Å × 30 Å was centred on the coordinates of the *α*-carbon of Asp 64 in the receptor molecule. The isomer ligand molecules and the receptor molecule were kept rigid during the experiments. Molecular docking experiments were performed using Autodock VINA [25] with the application's default settings. Each docking experiment produced 100 ligand-receptor pairs which were clustered using a RMSD cut-off of 5.0 Å. The pairs with the lowest binding energy were considered to have the best docking conformations. The results of each experiment were viewed using YASARA.

## 3. Results and Discussion

Natural products have been at the forefront of drug discovery and continue to provide viable pharmacophores and possible scaffolds for the development of potential drugs due to their structural diversity. Herbal plants used in traditional healing practices are often grown in areas with high UV exposure and no standard agricultural practices have been established. Therefore, it is expected that photoisomerisation reactions of bioactive compounds happen readily in such settings and so these active molecules may be compromised. As such, it is important that these environmental effects are taken into consideration when studying plant-derived drugs. In the current study, as previously mentioned, the effect of UV-induced geometrical isomerism of 3,5-diCQA was studied by considering structural (ligand) characteristics and biological activities of these isomers using* in silico* studies.

### 3.1. Ligand Stability

**Figure 1:**
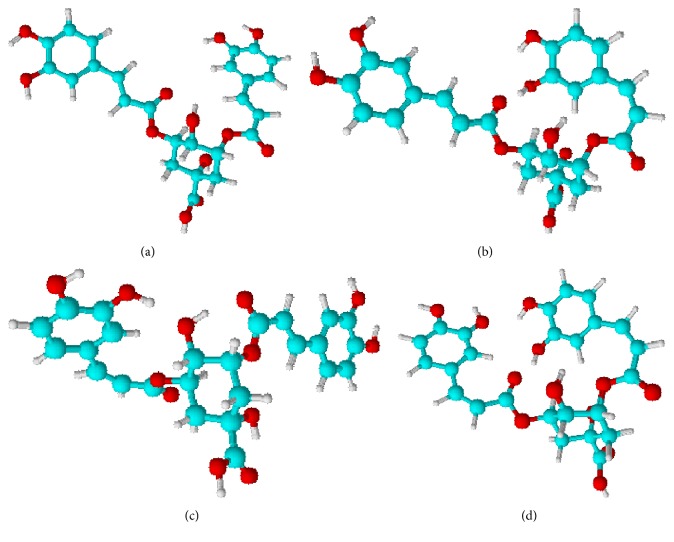
Geometry optimised structures of the geometrical isomers of 3,5-diCQA. (a) 3^*trans*^,5^*trans*^-diCQA, (b) 3^*cis*^,5^*trans*^-diCQA, (c) 3^*trans*^,5^*cis*^-diCQA, and (d) 3^*cis*^,5^*cis*^-diCQA.

Figure 1 shows the energy optimised structures of the 3,5-diCQA geometrical isomers. The geometry optimisation of the ligands suggests that the most stable isomer,* in vacuo*, is the 3^*trans*^,5^*cis*^-diCQA isomer followed by the 3^*cis*^,5^*trans*^-diCQA isomer; the naturally occurring 3^*trans*^,5^*trans*^-diCQA isomer had a relative energy of 1.758 kcal·mol^−1^ (Table 1). The low energy of the mono-*cis* isomers can be attributed to the fact that they have more intramolecular hydrogen bonds (IHBs) that act to stabilise their conformations than the 3^*trans*^,5^*trans*^-diCQA isomer. Additionally, these mono-*cis* isomers have minimal steric interactions as compared to the high energy 3^*cis*^,5^*cis*^-diCQA isomer; steric interactions have been shown to decrease the stability of molecules [26]. Due to the small difference observed in the relative energies of the isomers, it can be expected that these isomers coexist in plant material, with the exception of the 3^*cis*^,5^*cis*^-diCQA isomer (5.096 kcal·mol^−1^) which is likely to exist in a very minor concentration.

Though ligand stability in water and methanol solvent increases (results not shown), the structural conformations of the isomers remained the same. Since the main aim of the study was to determine differences in the binding interactions of the isomers to the INT enzyme, it suffices to use the* in vacuo* optimised structures to avoid solvent effects.

### 3.2. Docking Studies

**Figure 2:**
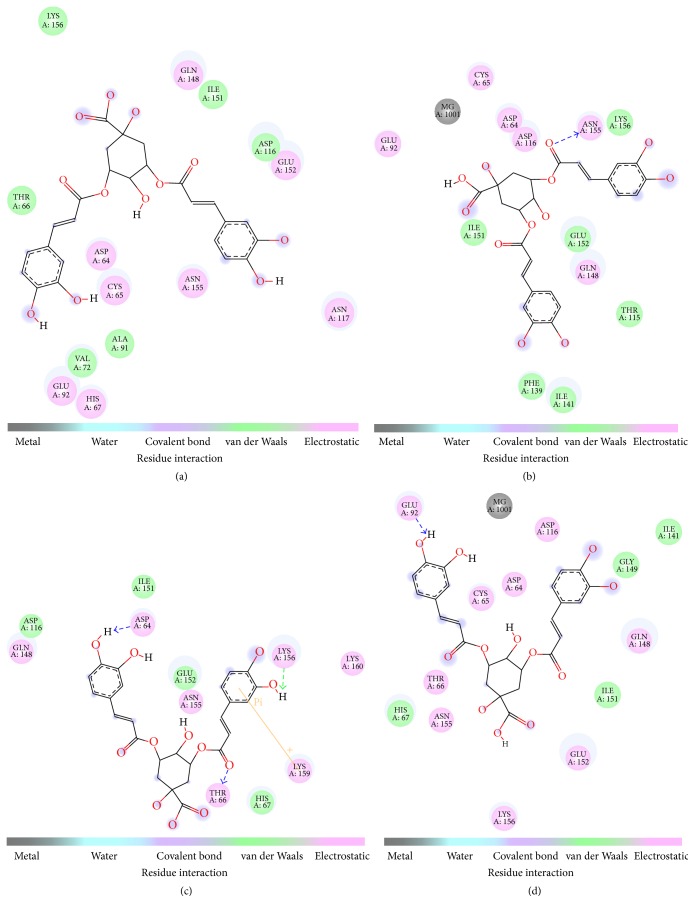
Two-dimensional representations of the interacting residues and the interaction type between HIV-1 INT and (a) 3^*trans*^,5^*trans*^-diCQA, (b) 3^*cis*^,5^*trans*^-diCQA, (c) 3^*trans*^,5^*cis*^-diCQA, and (D) 3^*cis*^,5^*cis*^-diCQA.

**Figure 3:**
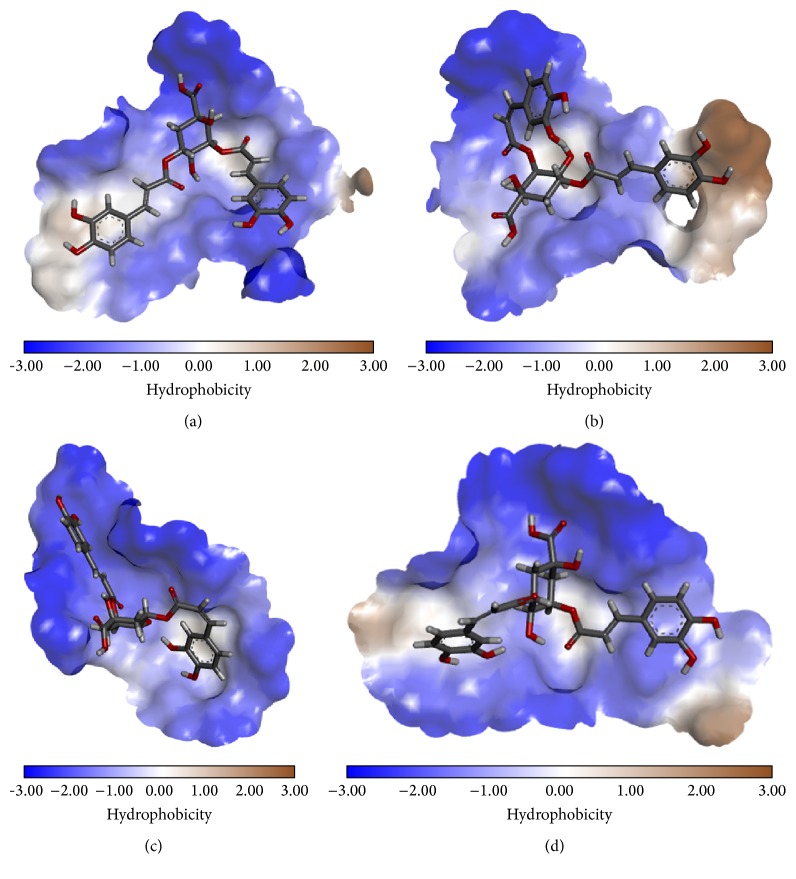
Three-dimensional maps of the hydrophobic interactions between HIV-1 INT and (a) 3^*trans*^,5^*trans*^-diCQA, (b) 3^*cis*^,5^*trans*^-diCQA, (c) 3^*trans*^,5^*cis*^-diCQA, and (d) 3^*cis*^,5^*cis*^-diCQA isomers.

The different geometrical isomers were found to exhibit very similar binding energies, which suggest that all the geometrical isomers are equally likely to bind to HIV-1 INT (Table 1). The docking results show that all the 3,5-diCQA isomers bind to the catalytic domain of the HIV-1 INT enzyme (Table 1; Figure 2) by electrostatic interactions and hydrogen bonds.  Figure 3 shows the localisation of the isomers in the catalytic domain of the enzyme and the hydrophobic characteristics of the catalytic domain. Apart from the hydrogen bonds, other weak interactions such as electrostatic interactions were also shown to exist (Figure 2). The interaction of these isomers with the INT enzyme through several forms of interactions is an indication that 3,5 diCQA is a viable drug candidate for the inhibition of this enzyme.

The amino acid residues that interact with the isomers, summarised in Table 1, include the catalytic triad residues ASP64, ASP116, and GLU152. This is consistent with experimental data that showed that the diCQAs interact with the conserved catalytic domain of retroviral integrase enzymes [7], which explains the potency of the diCQAs as HIV-1 INT inhibitors [27]. When comparing the hydrogen bonding interactions of the geometrical isomers, important differences were noted based on the type of contacting residues (Table 1). Here, the 3^*trans*^,5^*trans*^-diCQA and 3^*cis*^,5^*trans*^-diCQA isomers form hydrogen bonds with GLU152 while the 3^*trans*^,5^*cis*^-diCQA and the 3^*cis*^,5^*cis*^-diCQA form a hydrogen bond with ASP64. This suggests that caffeoyl moiety acylated on the 5 position of the quinic acid is essential for hydrogen bond interactions with the GLU152 and* cis*-isomerisation at this position diminishes this specific interaction. On the other hand, the caffeoyl moiety on the 3 position of the caffeoyl moiety is important for hydrogen bond interactions with the ASP64 and isomerisation (*cis*) abolishes this interaction. Taken together, a combination of these isomers would allow for interactions with the catalytic domain amino acid residues that are vital for enzyme functionality. Preliminary docking results with 3,4,5-tricaffeoylquinic acid suggest that it may possibly interact with all the residues that the individual 3,5-diCQA isomers interact with, which would explain its greater inhibitory activity against HIV-1 INT [8].

The lysine residues LYS156 and 159 are positioned in the catalytic domain of HIV-1 INT and are in close proximity to the active site residues. Previously, site directed mutagenesis of both these lysine residues resulted in the loss of disintegration activity [6]. Furthermore, photo-cross-linking experiments revealed that LYS 159 of the INT enzyme interacts with the viral DNA so as to orientate its phosphodiester bond close to the active site residues for further processing [6]. The naturally occurring 3^*trans*^,5^*trans*^-diCQA isomer and the UV generated 3^*cis*^,5^*trans*^-diCQA isomer interact with LYS 156 and not LYS 159 while the 3^*cis*^,5^*cis*^-diCQA isomer does not interact with any of these lysine residues (Figure 2). The 3^*trans*^,5^*cis*^-diCQA isomer interacts with both LYS156 and LYS159 and it furthermore interacts with LYS159* via* cation-pi interactions (Figure 2). In their theoretical studies, Nunthaboot et al. (2007) showed that the protonated form of LYS 159 is preferred when HIV-1 INT is complexed with the inhibitor, 5-CITEP [28]. This protonation state is likely to facilitate the cation-pi interactions that were observed between LYS159 and the catechol ring of 3,5-diCQA (Figure 2) [28]. The importance of cation-pi interactions for molecular recognition and in protein-ligand interactions has been well established [27, 29, 30]. Our study, therefore, highlights the potency of 3,5 diCQA as an inhibitor of this enzyme. This is well explained by these multiple forms of interaction which exist between the ligands and the enzyme, which suggests that the presence of all these four possible isomers can exhibit very strong synergistic interactions with INT enzyme. Moreover, Hu et al. suggested that contact with the active site residues (ASP64, ASP116, and GLU152) and the aforementioned lysine residues may hint to the mimicking of viral DNA by the dicaffeoylquinic acids as a mechanism of inhibition [13]. When c*is*-isomerisation occurs in both of the caffeic acid units, the ligand does not interact with any of these lysine residues [13].

Studies done on L-chicoric acid (L-CA) isomers showed a similar binding mode to that observed in this study with 3,5-diCQA geometrical isomers. In their study, Healy et al. observed that* cis-*isomerism in both caffeic acid arms (*s-cis/s-cis* L-CA) resulted in a conformation where both catechol units were well contained in the binding pocket which allowed for extensive hydrogen bonding to occur between the L-CA and the HIV-1 INT residues ASP116 and GLN148 [15]. With the* s-cis/s-trans* L-CA isomer, only one catechol ring formed a hydrogen bond with GLN148 and had extensive contact with GLU152 [15]. When compared with the known inhibitor 5 CITEP, the bidentate nature of L-CA and the diCQAs allows them to occupy the same region as the inhibitor and another adjacent pocket in the catalytic domain [13, 15].

**Figure 4:**
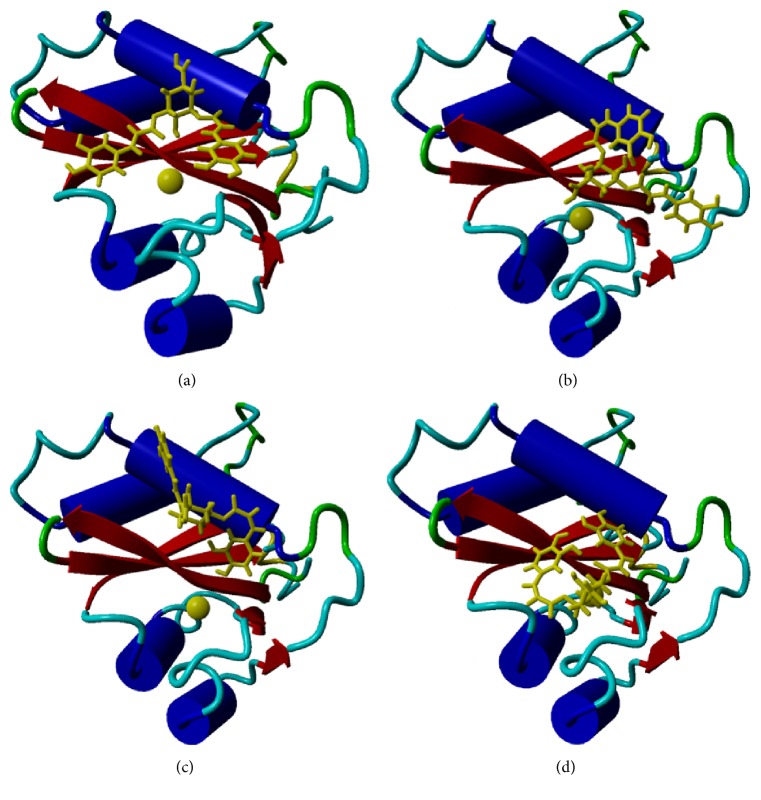
Ribbon structure of HIV-1 INT with the Mg^2+^ cofactor and the (a) 3^*trans*^,5^*trans*^-diCQA, (b) 3^*cis*^,5^*trans*^-diCQA, (c) 3^*trans*^,5^*cis*^-diCQA, and (d) 3^*cis*^,5^*cis*^-diCQA ligand. Molecular graphics were created using YASARA (http://www.yasara.org/) and POVRay (http://www.povray.org/).

Our results further indicate that* cis*-isomerisation at the caffeoyl moiety attached at the 3′ position of quinic acid seemingly allows the ligands to interact with the metal cofactor of the enzyme (Table 1, Figure 2). Here, 3^*cis*^,5^*cis*^-diCQA and 3^*cis*^,5^*trans*^-diCQA isomers can be seen to have some interaction with the Mg^2+^ cofactor (Table 1; Figure 4). Esposito and Craigie, when comparing INT activity in the presence of either manganese or magnesium, showed that viral DNA 3′ processing was optimal when magnesium was the metal cofactor [31]. Furthermore, metal interaction with 3,5-diCQA has been shown to be important for binding of the Human T-Lymphotropic Virus Type-1 (HTLV-1) INT enzyme which is homologous to HIV-1 INT [32]. In this work, it was shown that one Mg^2+^ ion interacting with INT and the ligand gave optimal energies of interaction between 3,5-diCQA and HTLV-1 INT [32]. However, in the current study and contrary to Peña and colleagues, 3^*cis*^,5^*cis*^-diCQA and 3^*cis*^,5^*trans*^-diCQA isomers were found to exhibit slightly lower affinity than those ligands that do not interact with the metal cofactor (Table 1).

Apart from* cis-trans* isomerism, other forms of isomerism of the diCQAs and related derivatives more especially on the quinic acid unit have been shown to affect the activity thereof. For instance, Jiang et al. showed that the addition of substituents on the quinic acid unit of the diCQAs decreased their antioxidant activity [33]. Furthermore, regional isomerism of the diCQA was also shown to affect their antioxidant activity [33]. Other instances where* cis-trans* isomerisation resulted in attenuated activity of biologically important molecules includes the 120-fold increase in the anti-*mycobacterium* activity of cinnamic acid when the* trans* isomer converts to the* cis* isomer [34]. Generally, the implications and effects of isomerisation of molecules also extend beyond the pharmaceutical and nutraceutical arena. As mentioned earlier, the* cis-trans* photoisomerisation of the tetrazoline oxime ether group of fungicides was shown to result in decreased efficacy when used on plants in the field compared to those in green houses; the consequence of which is reduced crop production [22]. The conversion of abscisic acid (a plant hormone used to prime stress resistance in plants) to its biologically inactive 2-*trans* isomer also places a threat on crop production [35]. Likewise, in the current study, geometrical isomerism of 3,5-diCQA attenuated the binding interactions of the ligand. Here, the 3^*cis*^,5^*trans*^-diCQA isomer causes a slightly lower binding affinity to HIV-1 INT and* cis*-isomerism of the caffeic acid arm at the 3′ position causes 3,5-diCQA to interact with the metal cofactor, highlighting the importance of studying geometrical isomerism of natural products. Although we have shown experimentally that all four isomers can exist [14], there is little evidence that all four exist in plants, such as in German chamomile [36] and* Achillea millefolium *L. [37], which have been exposed to natural light. As such, the negative results associated with the 3^*cis*^,5^*cis*^-diCQA isomer can be regarded as insignificant since its existence in nature is not well documented. Using antioxidative properties of both the positional and geometrical isomers, it was concluded that the presence of isomers in plants is an evolutionary strategy to maximize the existing pool of metabolites in order to exhibit stronger activities when needed [38]. In plant defence mechanism, the possible existence and involvement of* cis* of CQAs is also interesting [39]. As such, studies associated with geometrical isomers should be encouraged to fully elucidate their metabolic and pharmacological significance.

## 4. Conclusion

Using molecular docking, this study has shown that the geometrical isomers that result from the UV-irradiation of 3,5-diCQAs have important differences in their binding interactions with HIV-1 INT. These differences in the binding modes of the isomers do not, however, significantly affect the binding affinity of 3,5-diCQA to HIV-1 INT. The differences in their binding modes may point to a possible synergistic effect where a combination of all these isomers would cover a wider range of inhibitory activity than each individual isomer. Though it is improbable that these isomers would bind the catalytic site all at once, a ligand such as 3,4,5-tricaffeoylquinic may be a better inhibitor of HIV-1 INT. Further studies should focus on the preparation of these individual isomers in reasonable amounts so as to validate the findings of the current study experimentally. In light of these results, the agricultural practices governing the production of herbal plants for their anti-HIV activity may also need to be evaluated.

## Figures and Tables

**Table 1:** The relative energies for the 3,5-diCQA geometrical isomers and the results from the rigid docking studies with the HIV-1 integrase enzyme.

Isomer	Relative energy (kcal/mol)	Free binding energy (kcal/mol)	Contacting residues	H-bonded residues
3^*trans*^,5^*trans*^-diCQA	1.758	−9.332	**ASP64** CYS65 THR66 HIS67 VAL72 ALA91 GLU92 **ASP116** ASN117 GLN148 ILE151 **GLU152** ASN155 LYS156	CYS65 HIS67 **GLU152 **ASN155
3^*cis*^,5^*trans*^-diCQA,	1.320	−8.837	**ASP64** CYS65 GLU 92 THR 115 **ASP116** PHE139 ILE141 GLN148 ILE151 **GLU152** ASN155 LYS156 MG1001^*∗*^	CYS65 THR66 ASN117** GLU152**
3^*trans*^,5^*cis*^-diCQA	0.000	−9.173	**ASP64** THR66 HIS67 **ASP116** GLN148 ILE151 **GLU152** ASN155 LYS156 LYS159 LYS160	**ASP64 **GLU92 SER119 GLN148
3^*cis*^,5^*cis*^-diCQA	5.096	−9.082	**ASP64** CYS65 THR66 HIS67 VAL72 ALA91 GLU92 **ASP116** GLY118 GLN148 ILE151 **GLU152** ASN155 MG1001^*∗*^	**ASP64 **THR66 HIS67 GLN148

^*∗*^Divalent magnesium ion.
